# The Hard Way towards an Antibody-Based HIV-1 Env Vaccine: Lessons from Other Viruses

**DOI:** 10.3390/v10040197

**Published:** 2018-04-15

**Authors:** Oliver Ringel, Vincent Vieillard, Patrice Debré, Jutta Eichler, Hildegard Büning, Ursula Dietrich

**Affiliations:** 1 Georg-Speyer-Haus, Institute for Tumor Biology and Experimental Therapy, 60596 Frankfurt, Germany; ringel@gsh.uni-frankfurt.de; 2 Centre d’Immunologie et des Maladies Infectieuses (CIMI-Paris), Sorbonne Université, UPMC Univ Paris 06, INSERM U1135, CNRS ERL8255, 75013 Paris, France; vincent.vieillard@upmc.fr (V.V.); patrice.debre@aphp.fr (P.D.); 3 Department of Chemistry and Pharmacy, University of Erlangen-Nurnberg, 91058 Erlangen, Germany; jutta.eichler@fau.de; 4 Laboratory for Infection Biology & Gene Transfer, Institute of Experimental Hematology, Hannover Medical School, 30625 Hannover, Germany; buening.hildegard@mh-hannover.de; 5 German Center for Infection Research (DZIF), Partner Site Hannover-Braunschweig, 38124 Braunschweig, Germany

**Keywords:** HIV-1, vaccine, Env, broadly neutralizing antibodies, structure-based reverse vaccinology, epitope vaccine, vectored vaccine, adeno-associated viruses (AAV)

## Abstract

Although effective antibody-based vaccines have been developed against multiple viruses, such approaches have so far failed for the human immunodeficiency virus type 1 (HIV-1). Despite the success of anti-retroviral therapy (ART) that has turned HIV-1 infection into a chronic disease and has reduced the number of new infections worldwide, a vaccine against HIV-1 is still urgently needed. We discuss here the major reasons for the failure of “classical” vaccine approaches, which are mostly due to the biological properties of the virus itself. HIV-1 has developed multiple mechanisms of immune escape, which also account for vaccine failure. So far, no vaccine candidate has been able to induce broadly neutralizing antibodies (bnAbs) against primary patient viruses from different clades. However, such antibodies were identified in a subset of patients during chronic infection and were shown to protect from infection in animal models and to reduce viremia in first clinical trials. Their detailed characterization has guided structure-based reverse vaccinology approaches to design better HIV-1 envelope (Env) immunogens. Furthermore, conserved Env epitopes have been identified, which are promising candidates in view of clinical applications. Together with new vector-based technologies, considerable progress has been achieved in recent years towards the development of an effective antibody-based HIV-1 vaccine.

## 1. Introduction

Vaccines are the most efficient way to control epidemics caused by infectious pathogens. This is best exemplified by Edward Jenner’s discovery in 1796 showing that the infection of humans with a cowpox virus generates a crossprotective immune response against infection with human Variola virus, the cause of severe smallpox epidemics over centuries. Consequent worldwide vaccination programs under the umbrella of the World Health Organization (WHO) finally led to the eradication of smallpox in 1980 [1]. Since then, antibody-based vaccines against multiple human viruses causing pandemic diseases have been developed including influenza, hepatitis A and B, polio, papilloma, yellow fever, measles, etc. To really eradicate viral infections, besides clinical efficacy of the vaccine, there has to be the global political will and the economic power to implement such vaccines worldwide in all populations vulnerable to the respective virus [2]. Fortunately, increased scientific knowledge on viral pathogens and methodological progress in molecular techniques and viral vectors has accelerated vaccine development as recently exemplified by the development of a vaccine against the Ebola virus strain responsible for the 2014 outbreaks in West Africa [3]. Furthermore, a Coalition for Epidemic Prepardness Innovations (CEPI) consisting of academia, industrial companies, governments and public health agencies has been established recently to prioritize and to streamline the preclinical development of new vaccines against emerging potentially epidemic viruses such as Middle East respiratory syndrome (MERS), Lassa and Nipah [4].

**Figure 1:**
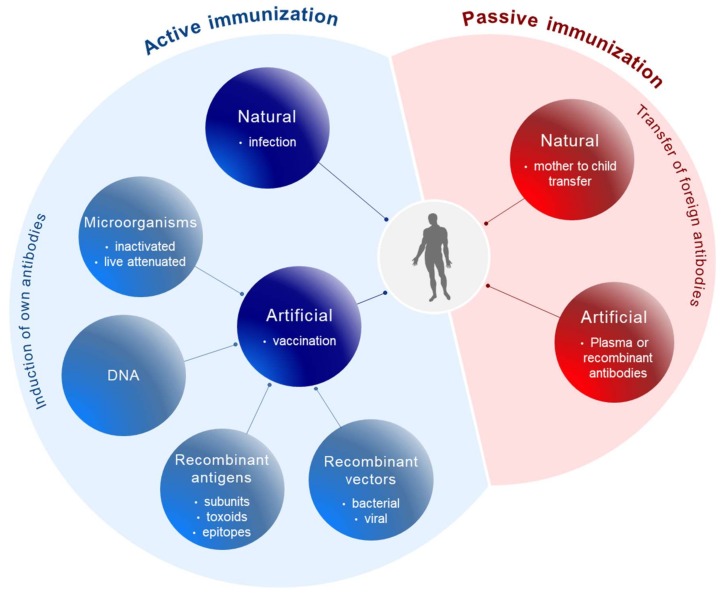
Principles of immunization. Active immunization (blue) involves the induction of the body’s own antibodies either after natural infection or after vaccination with antigen components derived from pathogens. Passive immunization (red) is based on the transfer of foreign antibodies against pathogens into the body for prophylactic or therapeutic purposes.

Conceptually, active and passive immunizations have to be distinguished (Figure 1). Active immunizations are based on antigen components of the respective pathogens, which upon introduction into the body, either by natural infection or by vaccination, generate after some weeks a “natural” long-lasting adaptive immune response able to neutralize and protect from infection.

**Figure 2:**
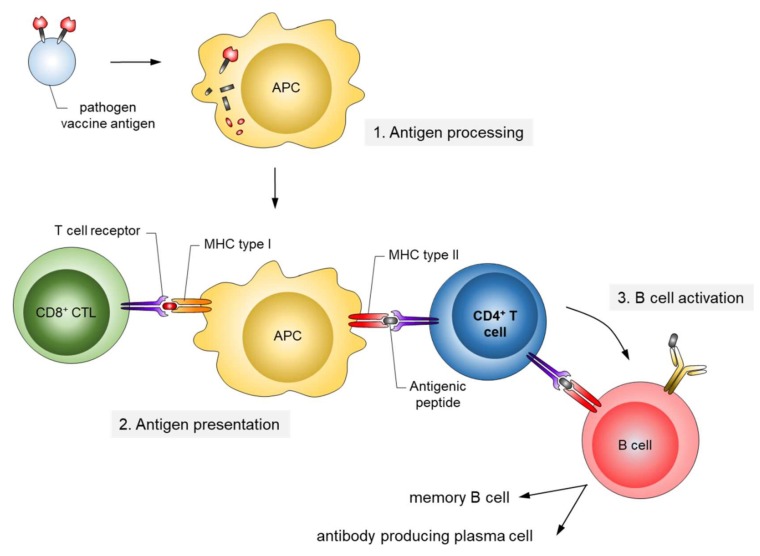
Mechanism of antigen presentation upon infection/active vaccination. Antigens ingested by antigen-presenting cells (APC) are processed by the proteasome, and antigenic peptides are presented on major histocompatibility complexes (MHC) types I and II. The peptide/MHC complexes are recognized by T cells. CD4-positive T helper cells (blue) play a central role in the activation of naïve B cells to differentiate into long-lasting antigen-specific antibody producing cells.

The corresponding antigens are processed by antigen-presenting cells (APC) to expose pathogen-specific peptides on major histocompatibility complexes (MHC) types I and II for activation of cytotoxic CD8^+^ and CD4^+^ helper T-cells, respectively (Figure 2). This natural immune response also includes the generation of long-lived memory B and T cells, which are rapidly activated and expanded upon the next encounter with the pathogen. In contrast, passive immunization consists of the direct administration of protective antibodies from external sources providing immediate, but short-lived, protection due to the limited half-life of antibodies (about three weeks). Passive immunizations can also occur naturally, i.e., by transfering antibodies from mother to child via the placenta or breast milk, or occur artificially by transfering purified natural or recombinantly expressed antibodies directly to the patients. Examples of infections prevented by passive vaccinations are rabies, tetanus and hepatitis A.

## 2. From Whole Pathogen and Subunit Vaccines to Epitope Vaccines

Different types of vaccines have been generated during the long history of vaccine development (Figure 1). Initially, whole microorganisms were used either in a live attenuated or in a killed/inactivated form. Due to their replicative capacity, live attenuated microorganisms are able to generate a very efficient long-lasting immune response upon vaccination; however, in immunocompromised patients attenuated microorganisms may revert to their initial replication potential and eventually cause disease. This was a matter of concern with oral polio vaccines [5,6] and the tuberculosis vaccine Bacillus Calmette-Guérin (BCG) [7]. For other virally related diseases like measles, mumps, rubella, yellow fever, etc. live attenuated viruses are still being used as vaccines. The rabies vaccine, in contrast, is based on an inactivated form of the virus.

For safety reasons and following technical advances allowing fast sequencing of the pathogens’ genomes and recombinant expression of antigens, subunit vaccines were also developed. These are mostly composed of surface components of the pathogens, as such antigenic components are often involved in the infection process, i.e., receptor-binding, and are also easily accessible for neutralizing antibodies [8]. Subunit vaccines may also involve modified toxins (toxoids) encoded by microorganisms to prevent their toxic pathogenic effects. Such vaccines are often being used in combination, i.e., combined toxoids for vaccination against diphteria, whooping cough (pertussis) and tetanus (DPT) [2]. An advantage of subunit vaccines in view of the diagnosis of viral infections is that vaccinated persons can be distinguished from infected persons at the serological level due to a restricted antibody response directed to the subunit vaccine.

For highly variable pathogens like influenza viruses, combinations of vaccine immunogens have to be used to cover the diversity of viral strains arising by antigenic drift and, occasionally, by new introductions from animal reservoirs [9,10,11]. This requires a continous monitoring of the circulating influenza virus strains [12] and, as recently described, computational prediction tools in order to be able to predict amino acid changes in avian influenza viruses, which may change the tropism for avian sialic acid receptors towards human sialic acid receptors in the next influenza season [13,14]. In parallel, attempts to achieve broad coverage of influenza viruses by a universal vaccine have also focussed on the identification of conserved epitopes in the virus surface proteins building the envelope spikes, which are essential for the infectivity of the viruses [15]. These spikes are composed of trimers of the viral hemagglutinin (HA), which is divided into the highly variable globular head (HA1) containing the receptor-binding site and the more conserved stalk domain (HA2) responsible for fusion [16]. Chimeric HA antigens and computationally optimized broadly reactive antigens (COBRA) based on multiple or ancestral HA sequences were generated and shown to protect in diverse animal models against influenza virus infection [17,18]. More recently, the identification of a whole series of monoclonal broadly neutralizing antibodies (bnAbs), facilitated by the introduction of new high-throughput techniques for antigen specific B-cell sorting from infected patients, and the downstream characterization of the corresponding target epitopes, contributed significantly to identify functionally and structurally conserved epitopes potentially suited as universal vaccines [19]. Although some of the bnAbs target the globular HA1 domain [20,21], most bnAbs target the more conserved though less immunogenic HA2 domain implicated in membrane fusion [22,23,24,25]. This region was further shown to induce antibodies with broad and potent antibody-dependent cellular cytotoxic (ADCC) activity, in contrast to the less broad neutralizing antibodies targeting the globular head of HA [10,26,27]. Thus, engineered mini-HA stem antigens were able to induce antibodies protecting mice in lethal heterologous challenge models [27]. Also, for other viruses like the severe acute respiratory syndrome-associated coronavirus (SARS-CoV), recombinantly expressed receptor-binding domains of the spike protein could protect from SARS-CoV challenge in animal models [28].

Even smaller spike components are epitope vaccines that should mimic conserved and functionally important domains in the otherwise highly variable surface proteins of the virus, which are essential for its infectivity. In that ideal case, the adaptive immune response generated upon vaccination with such epitopes should keep the virus in check, as escape mutations in these essential epitopes would severely compromise the infectivity of the virus. However, such epitopes often correspond to conformational epitopes, i.e., they are composed of protein stretches separated in the linear protein sequence, but juxtaposed in the tertiary protein structure. Even when the three-dimensional structure of the envelope components is known, the rebuilding of a conformational epitope in its natural functional form is still a challenge when separated from the protein context. Furthermore, the inclusion of additional vaccine-relevant domains like T cell epitopes, Toll-like receptor (TLR) agonists as adjuvants, and linkers in between these components complicates the expected vaccination outcome [29]. To better predict epitope vaccines based on antigen processing by proteasome transport to the endoplasmatic reticulum (ER) by TAP (antigen peptide transporter) and MHC binding, multiple in silico approaches have been developed [30,31]. Nevertheless, epitope-based vaccines are still in their infancy in the clinical vaccine trial pipeline [32].

With respect to infectious diseases, an epitope-based malaria vaccine (RTS, S = Mosquirix^TM^) has been developed, which is based on the expression of contiguous immunogenic epitopes from the predominant surface antigen of *Plasmodium falciparum* involved in the attachment to circumsporozoite protein (CSP) on liver cells. The epitopes were presented on the hepatitis B surface antigen (HBsAg) virus-like particle platform [33]. This vaccine was partially effective in a large phase III trial involving >15,000 children; however, protection was <30% and waned over time [34].

Also against influenza viruses, a universal epitope-based vaccine (Multimeric-001) was developed containing nine conserved B and T cell epitopes of influenza A and B viruses. This vaccine has recently entered clinical phase IIb [35], and phase III studies are planned by the producer company BiondVax. All epitopes are being expressed as a single recombinant protein of 50 kDa in *E. coli*.

## 3. HIV-1 Vaccine Development

### 3.1. Despite Effective Anti-Retroviral Treatment (ART), a Vaccine is Still Needed to Contain the Epidemic

**Figure 3:**
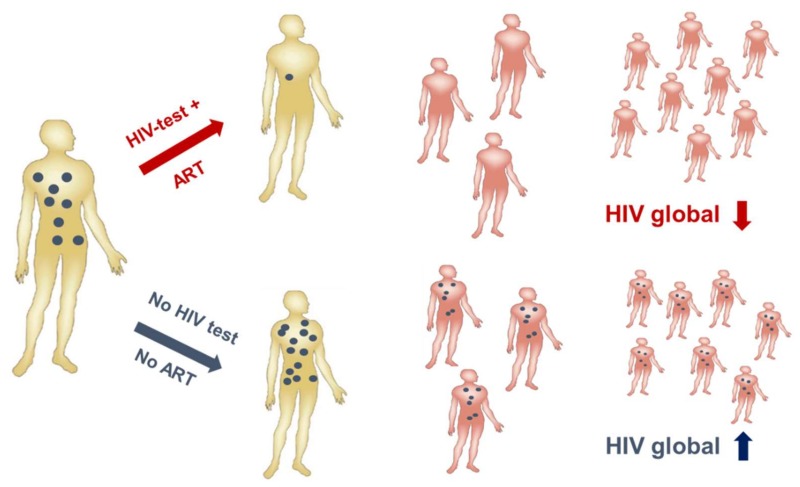
Human immunodeficiency virus (HIV) testing is essential to contain the HIV epidemic. A positive HIV test (upper panel) will be followed by the initiation of anti-retroviral treatment (ART) leading to reduced viremia (blue). Consequently, virus transmission is reduced/inefficient resulting in a global drop of HIV burden. Undiagnosed HIV infections will not be treated (lower panel) resulting in transmissions and a global rise in HIV infections. (Modified from Dietrich, [39]).

HIV-1 infection has spread worldwide, in particular in the last 40 years, and has caused one of the major recent epidemics affecting humanity. In total, more than 70 million people have been infected until now, and 35 million have died due to HIV/AIDS. Due to its predominantly sexual transmission, HIV-1 primarily infected younger generations, with devastating economic consequences for families and countries in the most affected regions such as Sub-Saharan Africa. A worldwide effort was initiated by the Joint United Nations Programme on HIV/AIDS (UNAIDS) and partners in 2014 to achieve the 90–90–90 agenda with the aim of diagnosing 90% of HIV infections, of which 90% should be on anti-retroviral treatment (ART) to result in 90% virally suppressed individuals in 2020 [36]. Currently, 36.7 million persons are living with HIV-1 worldwide. Although the number of new infections is declining in many countries due to a broader availability of ART, 1.8 million new infections were still registered in 2016 as well as 1 million HIV-related deaths. Furthermore, despite the huge success of ART in the treatment and prevention of HIV-1 infections worldwide, some regions like Eastern Europe and the Middle East have actually experienced a strong increase in new infections [37]. A further problem is that except for the HIV prevention programs recently applied in cohorts with high risk of HIV-1 infection, the vast majority of ART is given to persons with diagnosed HIV-1 infection status. However, even in higher income countries 20–30% of HIV-infected persons are not aware of their infection [38] and these persons are most likely to spread the virus among their contacts, as in the absence of ART higher viral loads increase the probability of HIV transmission (Figure 3). Therefore, intensification of HIV-1 testing is essential in order to be able to initiate ART early and to prevent further transmission of HIV-1.

The fact that many people are unaware of their infection by HIV-1 is also due to the nature of the virus itself: belonging to the genus *Lentivirus* within the family of *Retroviridae*, HIV-1 integrates its proviral DNA generated during reverse transcription of its RNA genome shortly after infection into the human chromosomes of infected cells, where it can persist for years in a latent state until reactivated by external stimuli. Furthermore, HIV-1 infection often does not cause obvious specific signs of HIV-related disease in the first years after infection, which is why these persons are unaware of their infection. Thus, HIV-1 can persist incognito with low-level replication in the absence of ART. Therefore, in order to prevent transmission, an HIV vaccine is still urgently needed. Effective vaccination would prevent transmission of HIV-1 from donors to recipients irrespective of their serological status. Furthermore, an effective vaccine could substitute the administration of antiviral drugs for prevention of infection in healthy people at risk.

### 3.2. The Bag of Tricks of HIV-1 to Evade the Immune System also Hinders Vaccine Development

**Figure 4:**
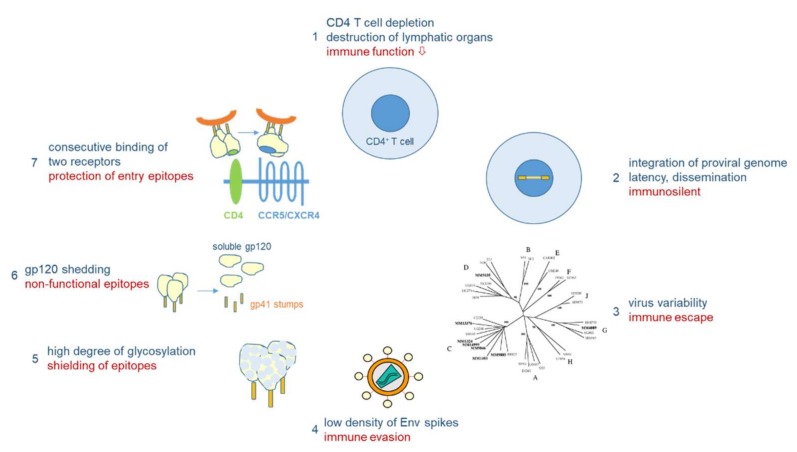
Immune evasion mechanisms of HIV-1. HIV-1 infects CD4^+^ cells, major players in adaptive immunity (1). Proviral integration results in lifelong persistence (2). Virus variability leads to immunological escape (3). Antigenicity is lowered by low number of HIV-1 envelope (Env) spikes (4) and extensive glycosylation (5). Non-functional Env epitopes are exposed through shedding of gp120 (6). Functional entry epitopes are protected through consecutive receptor interactions linked to conformational changes in Env (7).

Vaccine development against HIV-1 is one of the major challenges in medical research. Due to its high mutability (1–10 mutations/genome/replication cycle), HIV-1 has evolved a unique arsenal of tricks to evade the immune system (Figure 4). HIV-1 infects primarily CD4-positive T helper cells, which play a pivotal role in the adaptive immune system by activating B cells and cytotoxic T cells via co-stimulatory molecules and cytokine secretion. Infection of CD4-positive cells does not only kill the infected cells after virus production, but also kills uninfected CD4-positive cells via bystander mechanisms [40]. One such mechanism will be described below in Section 3.3. Early replication in lymphatic tissues (lymph nodes, gut), where CD4 target cells are densly packed together with antigen-presenting cells, destroys the architecture and functionality of the lymph nodes [41]. Thus, HIV-1 infection eliminates over time a very central cell type and organ of the immune system, finally resulting in acquired immunodeficiency (AIDS) and death in the absence of ART. However, this process takes time, as after integration HIV-1 can persist silently or at low replication levels. The latter allows the virus to mutate and to escape from the immune response of the host.

As outlined above for the influenza viruses, viral variability is the major problem for vaccine development. The variability of HIV-1 by far exceeds that of influenza viruses: in one single person infected by HIV-1, the virus type causing >95% of the worldwide HIV infections, the variability six years after infection corresponds to the global variability of all influenza viruses circulating in one year [42]. Besides the two HIV types and various subtypes of HIV-1 differing in about 40% in their Env amino acid sequences, HIV-1 continuously evolves within infected persons (driven by the high error rate of the viral reverse transcriptase), finally resulting in a “quasi-species” of related genomes with 5–10% divergence in a single patient. Besides fooling the immune system with a majority (>90%) of aberrant virus particles that are not functional, the high variability allows some mutant viruses to escape from the selection pressure imposed by the immune system or antiviral drugs in a functional form.

Other mechanisms of immune evasion of HIV-1 are mediated by the nature of the native Env spikes on the viral surface, which mediate infection through receptor binding and fusion, and which are the major targets for virus-neutralizing antibodies [43]. For HIV-1, the native Env spike is a trimer of three heterodimers, each composed of the outer envelope glycoprotein gp120 and the transmembrane glycoprotein gp41, which are both derived from the common precursor glycoprotein gp160 [44]. More than half of the mass of these glycoproteins is composed of sugars, which cover the outer surface of the spike, in particular in the variable loops of gp120, to reduce the immunogenicity of virus particles [45,46]. Furthermore, whereas hundreds of spikes are present on influenza viruses, HIV-1 particles usually contain only 14 spikes on average [47]. This low number is enough for infection, but minimizes the particle’s immunogenicity. In addition, the spikes are unstable, as the gp120 and gp41 components of the trimeric spike are non-covalently linked, resulting in shedding of the gp120 components. These monomeric gp120 proteins with a non-native conformation compared to their spike-associated counterparts, as well as the gp41 stumps left on the viral surface, mislead the immune system, which produces antibodies against immunodominant non-functional Env forms.

Finally, the trimeric Env spike does not have a fixed conformation, but is characterized by tremendous flexibility with a native closed form shifting towards more open conformations [48]. In addition, sequential binding to two different receptors is necessary to activate the fusion event between viral and cellular membranes, whereby binding to the first receptor, CD4, triggers conformational changes leading to the exposure of co-receptor binding epitopes, either binding to CCR5 or CXCR4 [49]. However, in this period the virus is already very close to the cellular membrane, so that many antibodies, in particular those against the membrane-proximal external region (MPER), can hardly interfere with infection.

The evasion tricks of HIV-1 do not only fool our immune system, but also did so with researchers involved in HIV vaccine research for decades. Thus, initial vaccine trials were based on soluble monomeric gp120 often derived from culture-adapted HIV-1 strains which, as we know today, due to its non-native conformation are not suited to induce broadly neutralizing antibodies (bnAbs), i.e., antibodies able to neutralize a broad spectrum of primary HIV-1 strains belonging to different subtpyes of HIV-1. At best, these vaccines could induce antibodies neutralizing autologous HIV-1 strains. Nevertheless, we learnt many lessons about how not to do it. Major advances in the field were obtained when soluble mimetics of the native trimeric Env spike (gp140 SOSIPs) were rationally designed based on structural and functional analyses [50,51,52,53]. These immunogens have been optimized during recent years, yet bnAbs against primary heterologous strains have not been induced so far upon vaccination, either in rabbits, monkeys or humans. At best, antibodies neutralizing the autologous primary strain were obtained [54,55]. However, an interesting finding was that upon immunization of camelids, broadly neutralizing nanobodies which correspond to the variable domain of heavy chain-only antibodies could be induced [56,57]. In a recent study, after the immunization of dromedaries with soluble trimeric subtype C proteins, two nanobodies with complementary neutralization patterns were obtained that neutralize 19 of 21 primary HIV-1 strains from a standard panel across different subtypes [58]. Interestingly, the immunization of cows with BG505 SOSIP also resulted in broadly neutralizing antibodies [59]. Camelids and cows both produce antibodies with longer HCDR3 (heavy-chain complementary determining regions), which is often a feature of bnAbs against HIV-1 [60].

In contrast to the difficulties encountered in the attempts to induce bnAbs against HIV-1 in vaccination studies, such bnAbs can be generated during natural chronic infection in a subset of HIV-1 positive patients [61]. Based on new technical developments like antigen-specific B cell sorting from patients, a plethora of bnAbs have been characterized in recent years (summarized in the bNAber database [62]). Their detailed characterization revealed peculiar features like a high degree of affinity maturation, long heavy chain complementarity-determining region (HCDR) 3 loops, and sometimes autoreactivity [63]. Furthermore, these bnAbs showed protective and therapeutic efficiency in humanized mouse models and in monkeys [64,65,66,67,68]. First, bnAbs in clinical trials were shown to reduce viremia and to delay viral rebound upon therapeutic applications [69,70,71,72]. Based on these promissing results further optimizations of SOSIP immunogens is ongoing with the aim of achieving the induction of bnAbs [73]. In particular, such immunogens should be able to engage naïve B cells which, after further sequential activation with related SOSIP constructs, should finally lead to the induction of high-affinity matured bnAbs [74,75,76].

### 3.3. Structure-Based Reverse Vaccinology and Epitope Vaccines for HIV-1

**Figure 5:**
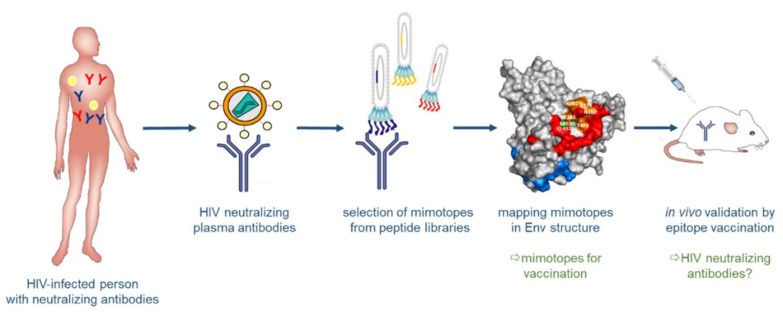
Structure-based reverse vaccinology. Broadly neutralizing antibodies (bnAbs) are generated in some HIV-positive persons. These bnAbs serve to select “mimotopes” from peptide libraries, which mimic the respective epitopes on Env. The selected epitopes are then analyzed computationally and structurally and evaluated for their capacity to induce, in turn, bnAbs upon vaccination.

The identification of bnAbs in patients chronically infected by HIV-1 and the recognition that these antibodies have protective and therapeutic efficacy not only in animal models, but also in first clinical trials, brought tremendous optimism into the field of HIV-1 vaccine development. Structural analyses of bnAbs in conjunction with Env antigens identified essential regions in the viral spike being targeted by bnAbs: these include the CD4 binding site, the apex of the variable loop 2 (V2), a high-mannose site in the variable loop 3 (V3) and the gp120/g41 interface [77,78,79,80,81]; additional sites comprise the MPER and the fusion peptide in gp41 (reviewed in [82]). The structural knowledge of these epitopes for bnAbs stimulated many studies focusing on the synthesis or expression of such epitopes as recombinant peptides to be used in turn as immunogens for the induction of bnAbs upon vaccination (Figure 5) [83,84,85]. This approach of structure-based reverse vaccinology or epitope-focused vaccine design is conceptually very elegant and has recently been proven to work in terms of inducing epitope-specific neutralizing antibodies against the respiratory syncytial virus (RSV) in vaccinated macaques, when the epitope was presented on computer-optimized flexible scaffolds [86]. However, it turned out to be more difficult for HIV-1 epitopes due to the intrinsic properties of the viral glycoproteins described above, in particular the high degree of glycosylation and structural flexibility, but also the co-evolution of Env and bnAbs over time resulting in “unmatched” late (affinity-matured bnAb with its generally heterologous Env target) and early (B cell-activating) epitope structures. Furthermore, binding of the bnAb to Env may induce structural alterations in Env (“induced fit”), so that the structure of the complex may potentially also represent a transitional state after Ab binding [87].

#### 3.3.1. Synthetic Peptide Mimics as Vaccine Candidates

Synthetic peptide strategies have been developed to mimic functionally important sites of the viral Env spike to be used as immunogens in active vaccination approaches [88]. As the CD4 binding site (CD4bs) of HIV-1 gp120 is essential for virus entry into CD4-expressing cells, the CD4bs is a promising target to interfere with virus entry. In fact, a range of bnAbs isolated from HIV-1 infected individuals were shown to recognize epitopes that overlap the CD4bs [89]. Therefore, synthetic mimics of the CD4bs were initially thought to be adequate immunogens to elicit a broadly neutralizing immune response similar to that of natural bnAbs which recognize the CD4bs.

In general, synthetic peptides—as compared to recombinantly generated peptides and proteins—are useful tools for the mimicry of protein sites, including epitopes for antibodies, since they can present natural protein fragments, and also allow for diverse chemical modifications, including the incorporation of a large range of non-proteinogenic amino acids [90]. Apart from extending the chemical and structural diversity presented by peptides, such modifications also increase the proteolytic stability of the molecules, enhancing their potential as drug candidates.

**Figure 6:**
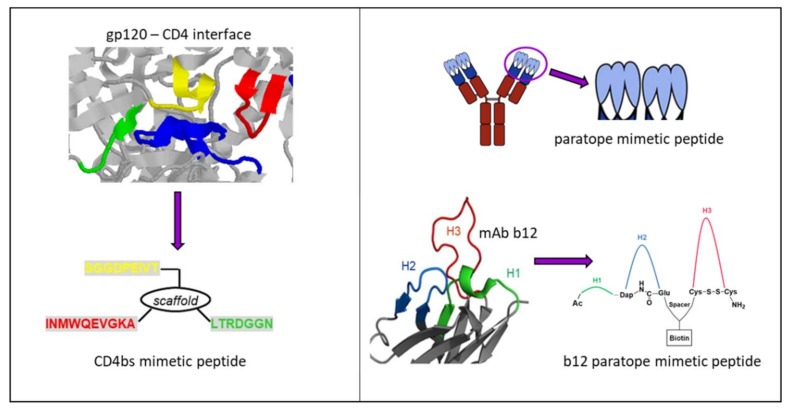
Peptides mimicking/targeting the complex CD4bs. (**Left**) Structure-based design of a CD4bs mimetic peptide (bottom) based on the crystal structure of a gp120-CD4 complex (top, pdb 1rzj). Fragments corresponding to the CD4bs are shown in red, yellow and green, respectively, and the CDR2-like loop of CD4 contacting gp120 is in blue. (**Right**) General design of antibody paratope mimetic peptides (top) and specifically for b12 (bottom) based on the b12-gp120 crystal structure (pdb 2ny7).

Based on the first published crystal structure of CD4–gp120 complexes [91], we have previously designed an assembled peptide (CD4bs-M) that presents three fragments of gp120, i.e., the CD4 binding loop, the β20/ β21 strand, and the β23 strand, which concertedly constitute the CD4bs [92] (Figure 6, left). Antisera obtained through the immunization of rabbits with this peptide specifically recognized gp120. Furthermore, the antisera competed with mAb b12, whose epitope overlaps the CD4bs of gp120 [93], for binding to gp120; however, the antisera were not found to be virus-neutralizing. Since in vitro binding assays had demonstrated that CD4bs-M inhibits the interaction of recombinant monomeric gp120 with soluble CD4 (sCD4), we had anticipated that CD4bs-M would be able to inhibit the gp120–CD4 interaction also in the context of HIV-1 infection, thus preventing infection of cells with HIV-1. Contrary to this expectation, we found that CD4bs-M strongly and specifically enhances the infection of target cells with HIV-1 [94]. Moreover, it was shown that this peptide also enables infection of otherwise unsusceptible cells with HIV-1. While it is likely that aggregation and fibril formation strongly contribute to the effects observed for CD4bs-M, we believe that the ability of CD4bs-M to trigger infection of CD4-negative cells points to additional mechanisms, such as the induction of conformational changes within Env, that may facilitate infection. This is based on our observation that CD4bs-M was shown to specifically interact with gp120 proteins from different HIV-1 strains, which was mediated by a fragment corresponding to the β20/β21 strand [94].

In an approach following the idea of reverse vaccinology, Zwick et al. identified a b12 mimotope, i.e., a peptide that is selectively recognized by the antibody, through screening of a phage-display library [95]. Unlike our CD4bs mimetic peptide, however, this b12 mimotope peptide was unable to elicit a gp120-cross reactive immune response when used as immunogen in mice [96]. HIV-1 Env regions other than the CD4bs of gp120, which have also been used as a template to design peptide immunogens, include the gp41 pre-hairpin intermediate, as well as the MPER of gp41. With the goal of eliciting neutralizing antibodies that target a transient viral entry intermediate of HIV-1 gp41, Bianci et al. used a range of peptides derived from the gp41 NHR region as immunogens for the vaccination of guinea pigs [97]. The resulting polyclonal antisera were shown to neutralize tier 1 HIV-1 isolates in vitro. In a structure-based reverse vaccinology approach, Serrano et al. designed a peptide immunogen based on the epitope of the broadly neutralizing antibody 2F5, which is located in the MPER of gp41 [98]. Immunization of rabbits with this peptide, in combination with liposomes, elicited a 2F5 cross-reactive immune response.

Synthetic peptide mimics have also been derived from antibody paratopes, i.e., the regions of an antibody specifically recognizing the corresponding antigen via its complementarity-determining regions (CDRs) on the surface of the variable domain of both heavy and light chains [99]. Molecules that mimic the CDRs of an antibody can, therefore, be expected to mimic the binding specificities of the antibody and, consequently, its effect in preventing the interaction of a disease-associated protein with its ligand [100] (Figure 6, right). We have recently reported on paratope mimetic peptides of the broadly neutralizing HIV-1 antibody b12 [101]. As described above, this antibody recognizes the CD4bs of the HIV-1 envelope glycoprotein gp120, and efficiently neutralizes HIV-1 infections in vitro and in vivo [102]. Based on the crystal structure of a b12-gp120 complex [103], we have designed an assembled peptide that presents the three heavy chain CDRs of b12 and, thus, the contact sites of the antibody for gp120 (Figure 6, right bottom). This b12 mimetic peptide was shown to bind gp120 at sub-micromolar concentrations, as well as to inhibit HIV-1 infection, demonstrating a functional mimicry of b12 by the paratope mimetic peptide. Furthermore, the b12 mimetic peptide was able to discriminate between gp120 from b12-susceptible and b12-resistant HIV-1, supporting the notion of functional mimicry of b12 by the peptide.

#### 3.3.2. Two Promising gp41 Epitope Vaccine Candidates

**Figure 7:**
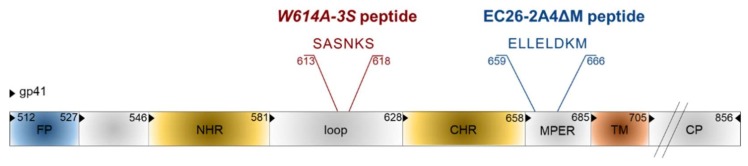
Epitope vaccine candidates in gp41. Functional domains are FP: fusion peptide, NHR: N-heptad repeat, CHR: C-heptad repeat, MPER: membrane proximal external region, TM: transmembrane domain, CP: cytoplasmic tail. Numbering is according to the HIV-1HXB2 amino acid sequence.

Besides the synthetic approach, epitope mimics for bnAbs from patient sera were also identified by the phage display technology [104]. Phage libraries presenting random peptide sequences [105,106] or shotgun-cloned larger HIV-1 Env fragments [107] were screened with immunoglobulins from HIV-positive sera with neutralizing activity. As bnAbs often target conformational epitopes, a special software was developed to analyze whether the selected linear mimotope sequences, without obvious linear homology to Env, could represent conformational epitopes on the Env surface [108]. Interestingly, some of the selected mimotopes could induce weak HIV-neutralizing antibodies in mice after vaccination with the mimotopes in the phage context, but also outside the phage, when coupled to different carriers [105,107,109]. In recent years, two interesting linear epitope sequences were identified in the gp41 transmembrane protein, EC26-2A4ΔM and W614A-3S (Figure 7), which will be discussed in more detail due to their clinical relevance for HIV epitope vaccine development. Gp41 also contains additional epitopes for neutralizing Abs like the first-generation mAbs 2F5 and 4E10 and the more potent bnAb 10E8, most of which localize to the MPER [110,111].

The original EC26-2A4 epitope was identified by screening an Env-tailored phage display library with plasma antibodies from an elite controller showing neutralizing activity for tier 2, i.e., the more neutralization-resistant, patient strains [107]. This epitope of 29 amino acids overlaps but clearly differs from the epitope of the first generation mAb 2F5 [110]. We could show that the EC26-2A4 epitope was, indeed, responsible for most of the neutralizing activity of plasma EC26, as (i) preabsorption of plasma with the epitope coupled to an affinity column reduced its neutralizing activity; (ii) plasma antibodies affinity-purified with the epitope had neutralizing activity; and (iii) immunization with the epitope coupled to different carriers induced neutralizing antibodies in mice [107,109]. As MPER antibodies like mAb 2F5 often show autoreactivity due to the partial integration of their epitopes in the membrane [111,112], the EC26-2A4 epitope was further optimized to determine the core epitope amino acids targeted by neutralizing antibodies from EC26, but avoiding autoreactivity with phospholipids [113]. This is important in view of vaccination studies, as autoreactivity may lead to the elimination of antibody-producing cells during B cell ontology or in the periphery [114]. Immunization of mice with the optimized peptide EC26-2A4ΔM in an Env DNA prime/peptide boost approach induced tier 1 neutralizing antibodies without cross-reacting with cardiolipin [113]. These experiments underline the suitability of the epitope EC26-2A4ΔM as a potential epitope vaccine candidate once the induction of antibodies neutralizing also tier 2 strains has been shown in more suited animal models. This is also supported by clinical data showing that a higher proportion (27%) of sera from a long-term non-progressor (LTNP) cohort (not treated by ART) reacted with the peptide compared to sera from a treated cohort (9%). Furthermore, in the treated cohort (close to 1000 sera), patients having EC26-2A4ΔM-specific antibodies at early and late stages after infection had a higher CD4 nadir, which according to the US Centers for Disease Control (CDC) determines the clinical stage of HIV infection [113,115]. Thus, antibodies targeting the EC26-2A4ΔM epitope may be beneficial for the patients.

**Figure 8:**
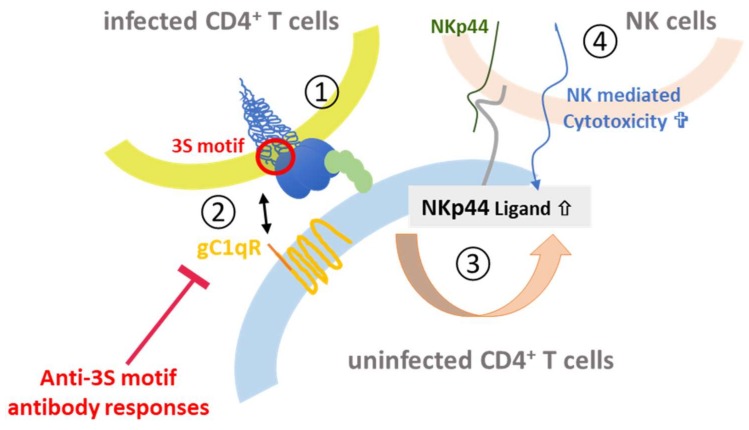
3S-peptide mediated depletion of CD4^+^ cells. Upon binding of Env on infected CD4^+^ T cells to the CD4 receptor (1) the 3S peptide (red) in gp41 interacts with the gC1qR complement receptor (orange) on uninfected CD4^+^ T cells (2). This leads to upregulation of the NKp44 ligand (gray) on the surface (3), which binds to NKp44 (green) expressed on NK cells (4) thereby mediating killing of the uninfected CD4^+^ T cell.

The other gp41 epitope, 3S, is most advanced with respect to clinical development. The highly conserved 3S motif interacts with gC1qR, a receptor for the globular form of the C1q complement factor. It thereby induces the expression of a cellular ligand of the activating NKp44 natural killer (NK) receptor rendering uninfected CD4-positive cells sensitive to NK cell lysis in HIV-1 positive patients [116,117,118]. The mechanism of action is composed of successive steps, which are outlined in Figure 8. The expression of NKp44 ligand is strongly correlated with disease progression and the decline of CD4 counts [116,117,118]. The 3S motif is characterized by its high level of conservation through HIV-1 strains of the different clades. Although these anti-3S Abs do not neutralize the virus, they are produced in humans at early stages of HIV-1 infection, and inversely correlate with CD4 cell-count decrease [119]. The results of a retrospective study including 244 untreated HIV-1 seropositve patients have revealed that the presence of high anti-3S Ab levels at the time of seroconversion is a factor predictive of CD4^+^ T cell protection during the first three years after infection, independently of the RNA and DNA viral loads [120]. These unique properties have served as the cornerstone in the development of a 3S vaccine strategy to prevent CD4^+^ T cell depletion and subsequent side effects, such as immune activation and inflammation. This concept was validated in non-human primate (NHP) models [121,122] and then successfully tested in a phase I/IIa clinical trial in HIV-infected patients under antiretroviral therapy, in collaboration with InnaVirVax [123].

The unique properties of the 3S peptide were further studied by an Alanine-walk through the 3S motif. A specific substitution at position 614, (called W614A-3S), was able to elicit bnAbs with cross-clade viral neutralization properties in mice immunized with the W614A-3S peptide. In a collaboration with Gabriella Scarlatti (Milan, Italy) we have observed that W614A-3S bnAbs were unparalleled in their magnitude, breadth and ability to confer their specific effects durably on tier 1 and tier 2 viruses from different clades (A, B, C and E) [124]. Moreover, two sets of data in rabbits and macaques have demonstrated the ability of the W614A-3S peptide to induce bnAbs in larger animal models. Altogether, these data demonstrate that W614A-3S is antigenic and, of interest, natural anti-W614A-3S Abs were also observed in about 4% of HIV-1 patients with high CD4 counts. After immune-purification against the W614A-3S peptide, Abs from serum of these patients were able to neutralize high titers of tier 2 viruses from clades B, C and E [124]. Interestingly, the presence of anti-W614A-3S bnAbs was strongly increased in untreated long-term non-progressor (LTNP) patients from the French ALT cohort (from the “Agence Nationale de Recherche sur le Sida” ANRS CO15), as compared to HIV progressor patients (25% vs. 4%), suggesting that these specific bnAbs are likely associated with a lack of disease progression. Their neutralizing capacity was inversely correlated with viral load and proviral DNA and associated with preservation of CD4 count [125]. CD4^+^ T cells from LTNP patients producing W614A-3S specific bnAbs could specifically recognize antigens from both HIV itself and recall antigens [125]. Conceivably, W614A-3S bnAbs participate in the protection of HIV-1 patients by continuous virological control resulting in maintenance of CD4^+^ T-cell counts and function. This study provides strong arguments that W614A-3S bnAbs contribute to the LTNP status, although in vivo immunization or passive-transfer experiments with purified W614A-3S Abs alone or in combination with other epitopes, like the optimized peptide EC26-2A4ΔM described above, will have to be investigated in the non-human primate model to determine their “protective” value.

### 3.4. Vectored Expression of bnAbs

Driven by the recent success of viral vectors in in vivo gene therapy, novel concepts emerged for confering protection against HIV infection or impairing re-infection. Besides vectorization of designer nucleases or recombinases for excision of HIV proviruses or removal of HIV co-receptors [126], viral vectors are considered as ideal tools for delivering the coding sequences of bnAbs, antibody-like-molecules (e.g., eCD4-Ig), immunoadhesins or entry-inhibiting peptides to name a few examples. Because of their excellent safety profile in human clinical trials, their low immunogenicity and high stability vectors derived from the adeno-associated viruses (AAV) have become particularly popular for this purpose [127,128]. AAV are non-pathogenic, replication-defective members of the *Parvoviridae* [129]. In AAV vectors, all viral open-reading frames are replaced by the transgene expression cassette comprising the gene of interest and its control elements. Albeit cloning of larger transgenes such as genes for bnAbs into AAV vector genomes is sometimes challenging due to the limiting coding capacity, a plethora of natural occuring serotypes and—more recently—of tailored or engineered capsids is available allowing a (nearly) free choice of target tissue and prime-boost vaccination schemes. Nonetheless, muscle has emerged as the main target tissue for the current approaches, resulting in continous release of bnAbs or other molecules employed for passive immunization from vector-transduced cells. This is a clear advantage in comparison to passive immunization with recombinant proteins, since their half life or bioavailibility requires frequent and, to be effective, life-long presence. In contrast to the latter, however, clinical experience with (AAV) vectors overexpressing bnAbs is limited so far to a single study. Initiation of that study is based on results from studies performed in humanized mice or in monkeys in which protection was demonstrated, as excellently summarized in a recent review [130]. A further advantage of using vectors for over-expression of bnAbs is the combined application of multiple bnAbs. The latter impressively decreases the risk of selecting immune-escaping variants, but is hard to realize by applying recombinant bnAbs due to cost issues.

**Figure 9:**
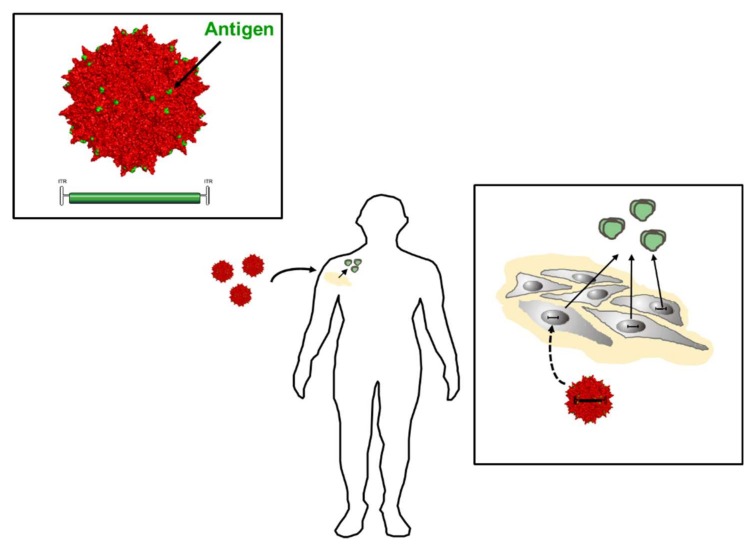
Adeno-associated viruses (AAV) vector-based vaccine approaches. Vectors deliver vector genomes that encode for antigens such as Env for continuous secretion from vector-transduced muscle cells. The choice of promoter defines the strength of antigen expression as well as the cell type that produces respective antigens. Capsid engineering technologies have allowed development of the next generation of vector-based vaccines, in which vector capsids (red) serve as scaffolds for antigen display (green dots). Thereby, a patient’s immune system is primed upon vector delivery through antigens already displayed on the capsid that at the same time functions as an innate immune stimulant. Following muscle transduction with these “prime-boost” vectors, the vector-encoded protein antigen is then secreted, providing the source for the antigenic boost (green Env trimers, right).

The concept of providing bnAbs to support the patient’s immune system is fascinating and has shown considerable promise, during the application of recombinant bnAbs directly in clinical trials or protection in monkeys, when expressed from AAV vectors. However, they are “foreign” for the host immune system and, thus, prone to anti-antibody induction. According to the seminal study by Martinez-Navio and colleagues, such antibodies are directed predominantly against the variable regions of the respective bnAbs [131]. Interestingly, the magnitude of the immune response strongly correlated with the maturation status of the antibody sequence, i.e., with the distance from germline, arguing that highly evolved antibodies are more easily recognized as foreign [131]. Besides this, other factors such as expression from muscle tissue (and not from B cells) may contribute. While use of an immune-suppression regime, avoidance of transgene expression in professional antigen-presenting cells, or induction of tolerance by targeting hepatocytes instead of muscle cells are considered as potential solutions [130], a novel generation of vectors may revive the concepts of inducing bnAbs by active immunization. Results of the ALVAC-HIV trail, which employed a prime-boost regimen using a canarypox vector encoding for HIV Env, gag and pro to prime the immune system followed by gp120 protein application as a boost to immunization, argued that indeed Env is the most promising target for active immunization approaches (Figure 9) [130].

Given the more recent developments in capsid engineering and the efficacy of antibody induction with virus-like particles [132] or fully competent vectors displaying epitopes or protein antigens at the capsid surface [133], one can envision priming of the immune system with an AAV vector displaying Env epitopes or gp120 on the viral capsid, which is used as scaffold for antigen display and at the same time as an inducer of innate immune responses. If these vectors encode at the same time for soluble Env, the immune system will receive a continous boost immunization intended to augment induction of bnAbs. In vivo studies have to show if this approach is feasible for HIV-1 Env antigens or if Env-intrinsic properties like immunosuppressive domains present in gp41 [134,135] may limit the immunogenicity.

## 4. Conclusions

Neutralizing antibodies have been shown to be the correlates of protection for many viral infections. Although bnAbs against primary heterologous HIV-1 have not yet been induced by the Env immunogens available today, the last few years have yielded crucial insights into the HIV-specific difficulties in vaccine development. The recognition that bnAbs identified from a subset of patients chronically infected by HIV-1 can protect from infection in animal models and reduce the viral load in HIV-positive patients in the first clinical trials emphasized the importance of bnAbs with respect to the goals that an HIV-1 vaccine should achieve. Furthermore, detailed characterization of the bnAbs has paved the way towards structure-based reverse vaccinology approaches to design superior immunogens with conserved features of native Env. In additon, these latest Env immunogens are currently being designed to be able to engage naïve B cells in order to stimulate the pronounced antibody-affinity maturation characteristic of many mature bnAbs against HIV-1. The future has to show if such immunogens are sufficient to generate bnAbs able to protect from infection by HIV-1 strains from all clades. Nevertheless, vaccines with less than 100% protection will also reduce the number of new infections and are worth pursuing. Meanwhile, vector-based expression of bnAbs is an option for achieving high expression levels in passive immunization approaches. This would avoid the high costs of recombinant expression of bnAbs; however, the development of anti-antibodies is still an issue. It is not yet known how broadly neutralizing nanobodies against HIV-1 will behave in clinics. Their small size and high solubility allows much better expression and also is expected to reduce the risk of anti-nanobody antibodies.
